# Elevated Soluble PD-L1 in Pregnant Women's Serum Suppresses the Immune Reaction

**DOI:** 10.3389/fimmu.2019.00086

**Published:** 2019-02-18

**Authors:** Mai Okuyama, Hidetoshi Mezawa, Toshinao Kawai, Mitsuyoshi Urashima

**Affiliations:** ^1^Division of Molecular Epidemiology, The Jikei University School of Medicine, Tokyo, Japan; ^2^Department of Pediatrics, The Jikei University School of Medicine, Tokyo, Japan; ^3^Division of Immunology, National Center for Child Health and Development, Tokyo, Japan

**Keywords:** PD-1, PD-L1, cord blood, pregnant woman, serum, MLC, IFN-γ

## Abstract

**Background:** Programmed death-ligand 1 (PD-L1) is expressed not only on some cancer cells, but also on the outer surface of placental syncytiotrophoblasts, which is assumed to induce maternal immune tolerance to fetal tissue via programmed death-1 (PD-1) receptors on T cells. Recently, levels of soluble forms of PD-L1 (sPD-L1) were reported to be higher in the serum of pregnant women (PW) than in non-pregnant women (non-PW). However, there have been no reports of the functional significance of PW's serum containing high sPD-L1 levels. Therefore, the aim of the present study was to clarify the role of sPD-L1 in the sera of PW as an immunosuppressive molecule by *in vitro* assays.

**Methods:** As a *post-hoc* analysis of our previous cohort study, 330 pairs of serum from PW during the third trimester and cord blood (CB) from paired offspring without major complications were examined. Serum levels of sPD-L1 and sPD-1 were measured by ELISA. On mixed lymphocyte culture (MLC), ^3^H-thymidine uptakes in the presence of PW's, offspring's, or non-PW's serum were compared. Peripheral blood mononuclear cells (PBMCs) were cultured in the presence of PW's serum stimulated with PHA, and then cytokine levels were measured in supernatants by multiple cytokine analysis with or without anti-PD-L1blocking antibody.

**Results:** The median sPD-L1 level was 8.3- and 6.9-fold higher in PW than in offspring and non-PW, respectively, whereas sPD-1 levels were lower in PW and offspring than in non-PW. On MLC, ^3^H-thymidine uptake in the presence of autoantigen was strongly reduced by co-culture with serum of both PW and offspring, compared with serum of non-PW. In contrast, uptake in the presence of alloantigen was moderately inhibited by PW's serum, whereas it was less suppressed by offspring's serum, compared with non-PW's serum. In the culture of PBMCs, tumor necrosis factor-α, interferon gamma, interleukin (IL)-2, and IL-4 levels were significantly higher in the presence of anti-PD-L1 blocking antibody than in culture not treated with antibody (all *P* < *0.05*) or culture treated with isotype control antibody (all *P* < *0.05*).

**Conclusion:** The levels of sPD-L1 are elevated in PW's serum, which may, at least in part, suppress maternal immunity.

## Introduction

Programmed death-ligand 1 (PD-L1) is expressed on some cancer cells to suppress anti-tumor immunity by interacting with the programmed death-1 (PD-1) receptor expressed on T cells (1). Indeed, blocking this interaction by administering monoclonal antibodies targeting either the PD-1 or PD-L1 molecule improves the prognosis of cancer patients (2, 3). By immunohistochemical staining with anti-PD-L1 antibody, PD-L1 was proven to be highly expressed on the outer surface of syncytiotrophoblasts on chorionic villi in placenta facing the maternal blood stream in the uterus, but not on the inner aspect of the syncytiotrophoblasts facing chorionic villous stroma, as well as not on cytotrophoblasts facing fetal blood vessels (4–6). It has long been the immunological paradox of pregnancy that, even though tissues of the fetus including the placenta express allogeneic paternal antigen in addition to autogenic maternal antigen, they are not rejected by maternal immune systems. Thus, this paradox may be explained at least in part by PD-L1 being highly expressed on placenta; like cancer cells, PD-L1 on the placenta suppresses T cells attached to the placenta and induces immune tolerance to fetal tissues via PD-1 receptors on T cells. In fact, blockade of the PD-1 pathway by anti-PD-L1 monoclonal antibody during pregnancy increased abortions in mice (7).

Both PD-L1 and PD-1 exist in membrane-bound form and induce local or peripheral immune tolerance by maintaining the quiescence of autoreactive T cells. Recently, soluble forms of PD-L1 and PD-1 (sPD-L1 and sPD-1), which are considered to be generated by proteolytic cleavage of the membrane-bound forms, have been detected in the serum of cancer patients (8, 9). ELISA has confirmed that sPD-L1 binds to PD-1 (10). In addition, a recent meta-analysis showed that a higher level of sPD-L1 is associated with worse overall survival of cancer patients (11), indicating that sPD-L1 may work as a systemic suppressor of anti-tumor immunity, in addition to local suppression of anti-tumor immunity by membrane-bound PD-L1 on cancer cells. In 2018, sPD-L1 levels were reported to be higher in the serum of pregnant women (PW) than in non-pregnant women (non-PW), and postpartum women (12). In contrast to sPD-L1, sPD-1 may block the interaction between PD-L1 and PD-1 on T cells by competitive inhibition (13), resulting in enhanced activity of autoreactive T cells and contributing to anti-cancer effects. These experimental results were further supported by both an animal model in which sPD-1-producing virotherapy successfully improved the prognosis of tumor-bearing mice (14) and clinical evidence in which non-small cell lung cancer patients with increased serum levels of sPD-1 showed prolonged survival (15). However, there have been no reports regarding serum sPD-1 levels in PW or the functional significance of elevated sPD-L1 levels in PW's serum. Therefore, the aim of the present study was to clarify the role of sPD-L1 in sera of PW as an immunosuppressive molecule by *in vitro* assays.

## Methods

### Study Design

As a *post-hoc* analysis, 330 pairs of PW and their offspring were randomly selected from our previous cohort study (16) conducted at Shiomidai Hospital, a general hospital in Kanagawa Prefecture, Japan. The inclusion criteria were: PW ≥ 20 years old at enrollment; lack of major complications, such as gestational diabetes mellitus, pregnancy-induced hypertension, pre-eclampsia, preterm labor, or the need for emergent cesarean section; and lack of high-risk fetal conditions, such as twins, intrauterine growth retardation, and congenital malformations. PW were enrolled from June 2011 to September 2012. Because sPD-L1 levels vary with age, 20 commercial serum samples from non-pregnant healthy women in their twenties and thirties were initially purchased for use as age-matched controls. To compare serum sPD-L1 levels among non-PW with known smoking status, 21 commercial serum samples from non-pregnant healthy women were also purchased: non-smokers, *n* = 7; past smokers, *n* = 7; and current smokers, *n* = 7.

### Ethics

The study protocol was approved by the ethics committee at the Jikei University School of Medicine, the clinical study committee at Jikei Hospital, and the institutional review board at Shiomidai Hospital. Clinical data and samples were anonymized immediately after their collection at birth in a non-linkable fashion. Data monitoring was performed in the Division of Epidemiology, the Jikei University School of Medicine, with all data monitored and fixed by HM, who did not participate in ELISA measurements or statistical analyses. All women provided their written, informed consent. The serum samples used for controls were purchased from Tokyo Future Style, Inc. (Tsukuba, Ibaraki, Japan).

### Measurement of sPD-L1 and sPD-1 Levels

Serum samples were collected from PW at 34 weeks of gestation. The offspring's serum (5–10 mL) was sampled from the placental side after placental delivery at birth. The serum samples were stored at −80°C prior to use. Serum levels of sPD-L1 and sPD-1 were measured by MO, using ELISA kits from Abcam (Cambridge, MA, USA) and RayBiotech (Norcross, GA, USA), respectively, according to the manufacturers' protocols. Each sample was tested in triplicate for sPD-L1 and in duplicate for sPD-1, with the medians used for analysis. The lower detection limits for ELISA were 3.9 pg/mL for sPD-L1 and 20 pg/mL for sPD-1. The upper detection limits for ELISA were 1,300 pg/mL for sPD-L1 and 6,000 pg/mL for sPD-1.

### Mixed Lymphocyte Culture

Reactions of lymphocytes in the presence of either autoantigen or alloantigen were measured by ^3^H-thymidine uptake using a mixed lymphocyte culture (MLC) assay system at SRL Inc (Hachioji, Tokyo, Japan). Briefly, peripheral blood mononuclear cells (PBMCs) were obtained from three healthy male volunteers, named A, B, and C. For the MLC assay with autoantigen, fresh PBMCs were co-cultured with 13-Gy-irradiated PBMCs from the same donor in three patterns, i.e., fresh A—irradiated A, fresh B—irradiated B, and fresh C—irradiated C. For the MLC assay with alloantigen, fresh PBMCs were co-cultured with 13-Gy-irradiated PBMCs from different donors in four patterns, i.e., fresh A—irradiated B, fresh A—irradiated C, fresh B—irradiated A, and fresh C—irradiated A. These cells were cultured for 5 days with RPMI1640 and 20% of either a mixture of serum samples from PW, offspring, or non-PW, randomly selected from the cohort of this study. Each kind of serum was a mixture of at least 10 samples. Cells were pulsed with ^3^H-thymidine during the last 17.5 h of incubation and counted in a liquid scintillation counter (Microplate Scintillation and Luminescence Counter; Perkin Elmer, Inc, Waltham, MA, USA). This MLC experiment was repeated for three sets using the same serum samples.

### Cytokine Assay

PBMCs were obtained from a healthy female volunteer. Then, 2.0 × 10^4^ PBMCs per well in 96-well U-bottom plates were co-cultured with RPMI1640 and a mixture of serum samples derived from at least 10 PW randomly selected from the cohort of this study. The serum concentration was brought to 5%, and treated with 5 μg/ml of phytohemagglutinin (PHA) (J-CHEMICAL, Inc., Chuo-ku, Tokyo, Japan) for stimulation. Samples treated with anti-PD-L1 blocking antibody (5 μg/ml) (Monoclonal Antibody MIH1, Functional Grade, eBioscience, San Diego, CA, USA), isotype control (5 μg/ml) (mouse IgG1 kappa Isotype Control, Functional Grade, eBioscience), and samples without antibody were cultured according to the method of Andorsky et al. (17). After 72 h of culture, supernatants were harvested from culture, and tumor necrosis factor-α (TNF-α), interleukin 6 (IL-6), interferon gamma (IFN-γ), IL-2, and IL-4, as markers of broad immune responses, were measured by quantitative multiplex detection using the Human Magnetic Luminex Screening Assay (R&D System, Minneapolis, MN, USA), since these cytokines were reported to be secreted from PBMCs stimulated by PHA (18).

### Statistical Analyses

Because the skewness test showed that sPD-L1 levels and sPD-1 levels were not normally distributed, their levels were compared among control non-PW, PW, and CB using the Kruskal-Wallis rank test (KW test). If the result of the KW test was significant, then the Mann-Whitney test was used to compare between two groups. Spearman's rank correlation, represented as rho, with linear regression was used to quantify the strengths of associations between two continuous variables: rho ≥0.4, strong; 0.4> rho ≥0.2, moderate; and rho <0.2, weak. The test for trends across ordered groups developed by Cuzick (19) was used to examine the relationships between anthropometric measurements at birth and quartiles of sPD-L1. Multivariate analysis with linear regression was performed to adjust for potential confounders of the associations between anthropometric data and sPD-L1 quartiles, gestational weeks, and sex of offspring. For ^3^H-thymidine uptake on MLC, as well as production levels in cytokine assays, the KW test and the Mann-Whitney test were used to compare among three groups and between two groups, respectively. Stata version 14.0 software (StataCorp, College Station, TX, USA) was used for all analyses. Values of *P* < 0.05 were considered significant. These analyses were not corrected for multiple comparisons.

## Results

### Participants' Characteristics

In a *post-hoc* manner, 330 pairs of PW and offspring were analyzed in this study; the demographic and clinical data are shown in Table 1. Moreover, 20 serum samples from non-PW were used as controls.

**Table 1:** Participants' characteristics.

	**Pregnant women *n* = 330**	**Offspring *n* = 330**	**Non-pregnant women *n* = 20**
Age (y) mean (*SD*)	32 (5)	–	29 (7)
Female ratio (%)	100%	48%	100%
Body height (cm) mean (*SD*)	158.6 (5.2)	48.7 (1.9)	
Body weight (kg) mean (*SD*)	52.9 (8.1)	3.057 (0.410)	
Weight change (kg) mean (*SD*)	8.6 (3.6)		
Gestational weeks mean (*SD*)	38.7 (1.3)		
Apgar score, median (25–75%)			
1 min		9 (8–9)	
5 min		9 (9–10)	

### Serum Levels of sPD-L1 and sPD-1 Among PW, Offspring, and Non-PW

**Figure 1:**
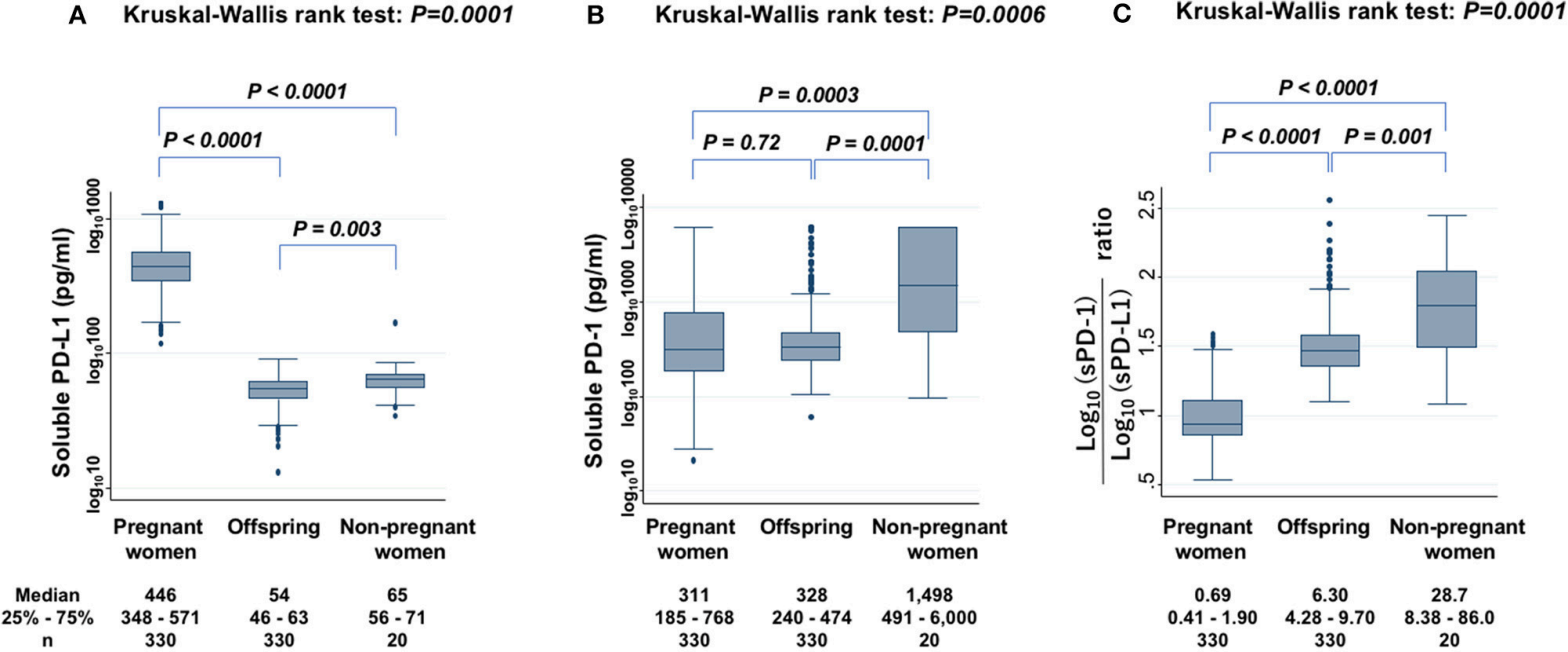
Levels of sPD-L1 **(A)**, sPD-1 **(B)**, and the ratio of sPD-1 to sPD-L1 **(C)** in serum from PW, offspring, and non-PW controls. The Kruskal-Wallis rank test (KW test) was used for comparisons among three groups. The Mann-Whitney test was used to compare two groups. Values were transformed by the common logarithm (log_10_) prior to analysis and are shown in the graph, although the median and 25−75th percentiles are presented as absolute values.

Serum levels of sPD-L1 were first compared among PW, offspring, and non-PW to find significant differences (KW test: *P* = 0.0001) (Figure 1A). The median level of sPD-L1 was 8.3-fold higher in the serum of PW than in offspring (*P* < 0.0001) and 6.9-fold higher in PW than in non-PW (*P* < 0.0001). The median level of sPD-L1 was lower in offspring than in non-PW (*P* = 0.003). Then, serum sPD-1 levels were compared among the three groups to find significant differences (KW test: *P* = 0.0006) (Figure 1B). In the serum of non-PW, the median level of sPD-1 was 4.8-fold higher than in serum of PW (*P* = 0.0003), and 4.6-fold higher than in serum of offspring (*P* = 0.0001). However, the sPD-1 level was not significantly different between PW and offspring (*P* = 0.72). The median sPD-1/sPD-L1 ratio of non-PW was 41.6-fold higher than in PW (*P* < 0.0001) and 4.6-fold higher than in offspring (*P* = 0.001), and the ratio of offspring was 9.1-fold higher than in PW (*P* < 0.0001) (Figure 1C).

### Collinearity of sPD-L1 and sPD-1 Serum Levels Between PW and Paired Offspring

**Figure 2:**
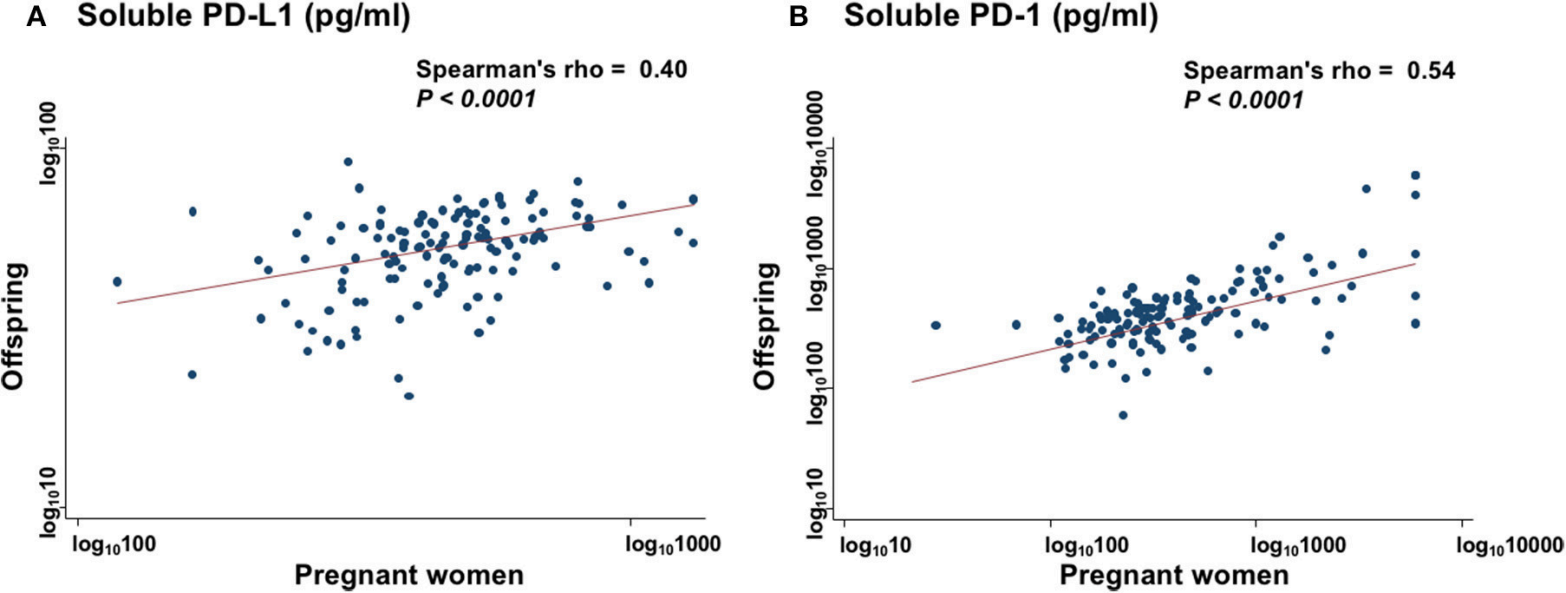
Collinearity levels of sPD-L1 **(A)** and sPD-1 **(B)** in serum between PW and matched offspring. Spearman's rank correlation with linear regression was used to quantify the strength of association; sPD-L1 values were transformed by common logarithm (log_10_) prior to analysis.

The sPD-L1 levels in PW had a strong positive association with those in offspring (Spearman's rho = 0.40; *P* < 0.0001) (Figure 2A). The sPD-1 levels in PW also had a strong positive association with those in offspring (Spearman's rho = 0.54; *P* < 0.0001) (Figure 2B). In contrast, there were no associations between sPD-L1 and sPD-1 levels in serum of PW and of offspring.

### Mixed Lymphocyte Culture

**Figure 3:**
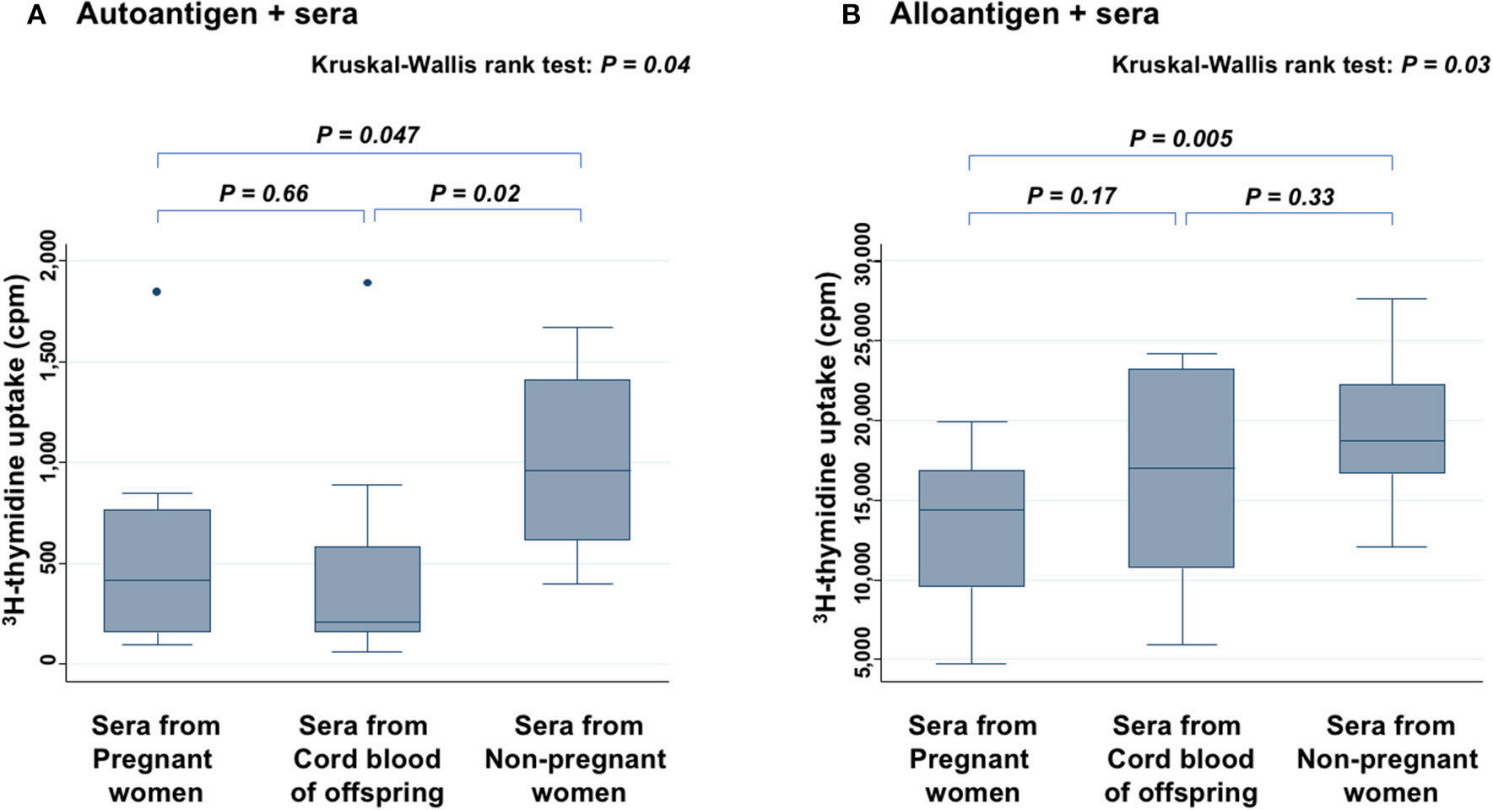
Mixed lymphocyte culture assays in the presence of autoantigen **(A)** and alloantigen **(B)**. Levels of ^3^H-thymidine uptake of peripheral blood mononuclear cells (PBMCs) are compared among sera from PW, offspring, and non-PW. The Kruskal-Wallis rank test (KW test) was used for comparisons among three groups. The Mann-Whitney test was used to compare two groups.

Level of ^3^H-thymidine uptake in the presence of autoantigen (Figure 3A) and alloantigen (Figure 3B) were compared among three groups: PW, offspring, and non-PW. In the presence of autoantigen, median ^3^H-thymidine uptake was strongly reduced by co-culture with serum from both PW (57% reduction; *P* = 0.047) and offspring (78% reduction; *P* = 0.02), compared with that of non-PW. On the other hand, there was no difference in the uptake between sera from PW and offspring. In the presence of alloantigen, ^3^H-thymidine uptake was moderately reduced by co-culture with serum from PW (23% reduction; *P* = 0.005), and not significantly reduced by co-culture with offspring's sera (*P* = 0.33), compared with that of non-PW.

### Cytokine Assay

**Figure 4:**
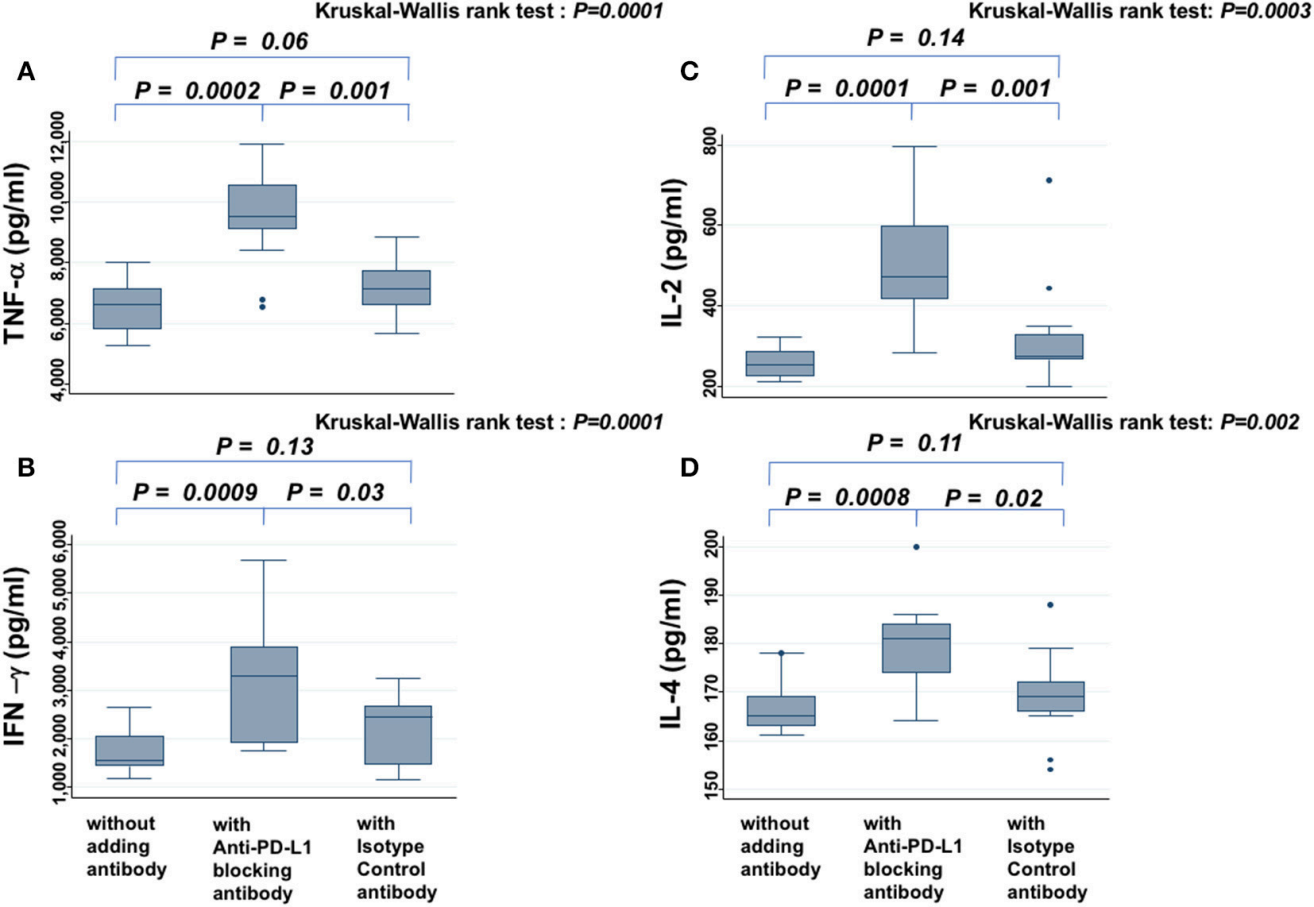
Cytokine assay. Levels of TNF-α **(A)**, IFN-γ **(B)**, IL-2 **(C)**, and IL-4 **(D)** secreted from PBMCs in the presence of PHA and PW's serum are compared among samples without antibody, those treated with anti-PD-L1 blocking antibody, and those treated with isotype control antibody. The Kruskal-Wallis rank test (KW test) was used for comparisons among the three groups. If there was a significant difference, the Mann-Whitney test was used to compare two groups.

In the presence of PW's sera, anti-PD-L1 blocking antibody increased the secretion of TNF-α (Figure 4A), IFN-γ (Figure 4B), IL-2 (Figure 4C), and IL-4 (Figure 4D), significantly more than samples without antibody or those treated with isotype antibody (all *P* < 0.05). On the other hand, the levels of these cytokines did not show significant difference between samples without antibody and those treated with isotype antibody. There was a similar tendency for IL-6, but it was not significant (data not shown).

### Levels of sPD-L1 and Smoking Status in PW and Non-PW

**Figure 5:**
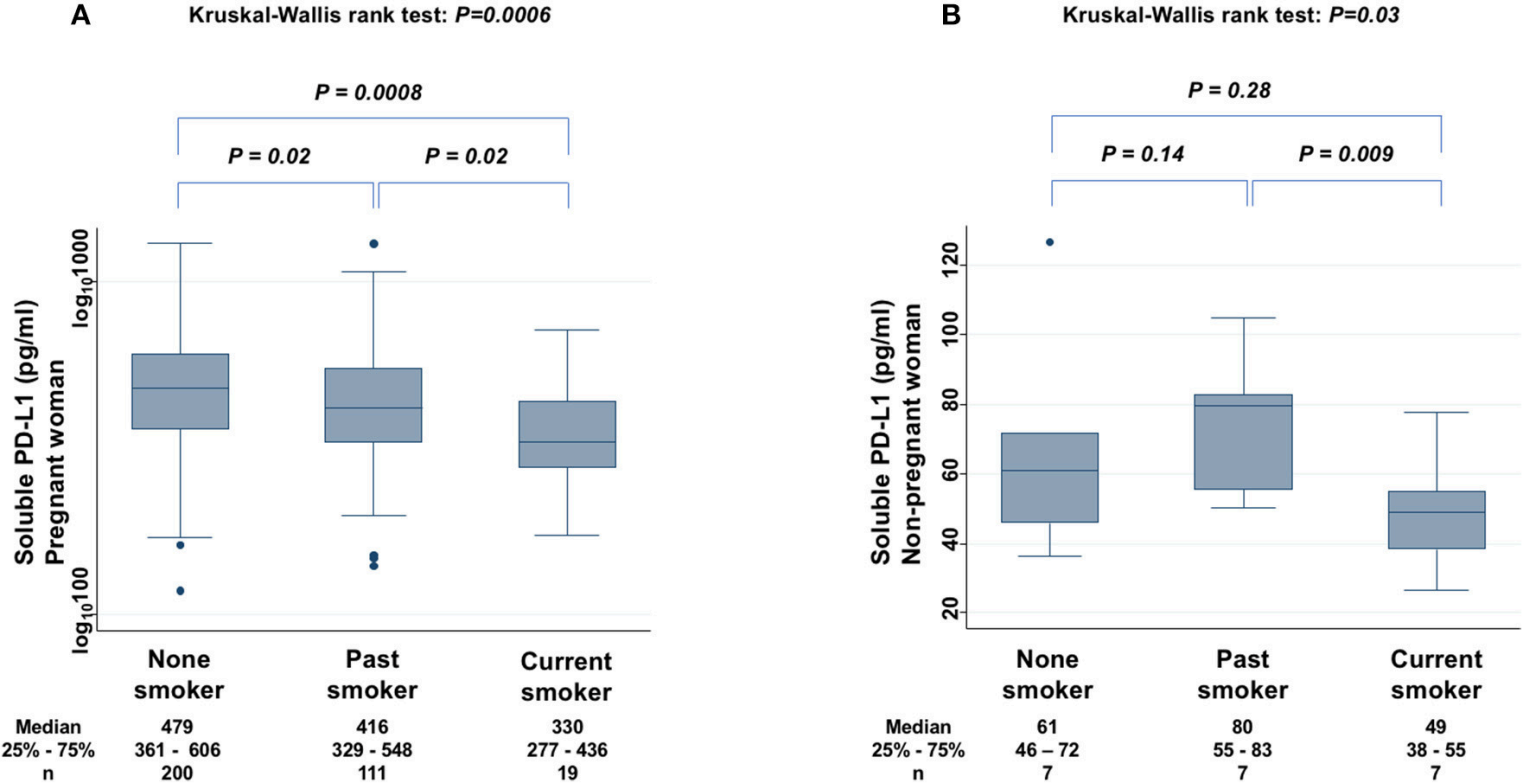
Relationship between sPD-L1 levels and smoking status in PW **(A)** and in non-PW **(B)**. The Kruskal-Wallis rank test (KW test) was used for comparisons among three groups: non-smoker, past smoker, and current smoker. The Mann-Whitney test was used to compare two groups; sPD-L1 values were transformed by the common logarithm (log_10_) prior to analysis and are shown in the graph **(A)**, although the median and 25−75th percentiles are presented as absolute values.

The levels of sPD-L1 were compared among non-smokers, past smokers, and current smokers among PW to find significant differences (KW test: *P* = 0.0006) (Figure 5A). The sPD-L1 levels were 13 and 31% lower in past and in current smokers, respectively, than in non-smokers. Serum levels of sPD-L1 were also measured among non-PW (KW test: *P* = 0.03) (Figure 5B). In particular, the sPD-L1 levels were 27% lower in current smokers than in non-smokers plus past smokers (Mann-Whitney test: *P* = 0.03), whereas there was no significant difference in sPD-L1 levels between non-smokers and past smokers.

### Offspring Anthropometric Measurements and sPD-L1 Levels in PW

Offspring anthropometric measurements at birth were compared with serum sPD-L1 levels in PW as both quartiles and continuous variables.

The median body weight at birth in the highest sPD-L1 quartile (Q4) was 270 g heavier than in the lowest quartile (Q1). Thus, there was a significant trend for increased body weight with higher levels of sPD-L1 (trend test, *P* < 0.0001) and a moderate Spearman's rho (0.24, *P* < 0.0001) (Supplementary Figure 1). On multivariate adjustment with gestational weeks and offspring's sex, body weight remained a significant factor.

The median body height of the highest quartile (Q4) of sPD-L1 was 0.6 cm taller than that of the lowest quartile (Q1). There was thus a significant trend for increased body height with higher levels of sPD-L1 (trend test, *P* < 0.0001) and a weak Spearman's rho (0.13, *P* = 0.02) (Supplementary Figure 2). On multivariate adjustment with gestational weeks and offspring sex, body height remained a significant factor.

The median head circumference of the highest quartile (Q4) of sPD-L1 was 0.4 cm longer than that of the lowest quartile (Q1). There was thus a significant trend for increased head circumference with higher levels of sPD-L1 (trend test, *P* = 0.009) and a weak Spearman's rho (0.15, *P* = 0.007) (Supplementary Figure 3). On multivariate adjustment with gestational weeks and offspring sex, head circumference remained a significant factor.

The median chest circumference of the highest quartile (Q4) of sPD-L1 was 0.5 cm longer than that of the lowest quartile (Q1). There was thus a significant trend for increased chest circumference with higher levels of sPD-L1 (trend test, *P* < 0.0001) and a moderate Spearman's rho (0.21, *P* = 0.0001) (Supplementary Figure 4). On multivariate adjustment with gestational weeks and offspring sex, chest circumference remained a significant factor.

## Discussion

In the present study, the median serum sPD-L1 level in PW during the third trimester was high, 8.3-fold higher than in offspring and 6.9-fold higher than in healthy age-matched non-PW controls. Although serum levels of sPD-L1 were reported to increase throughout gestation (12) when blood samples from 30 PW were collected, this increase of sPD-L1 in PW was reconfirmed by expanding the sample size to 330 PW, and their offspring's CB was found to have lower levels of sPD-L1 than the serum of non-PW. In contrast to sPD-L1, sPD-1 levels were lower in PW and offspring than in non-PW. Opposite to sPD-L1, sPD-1 was reported to competitively inhibit the interaction between PD-L1 and PD-1 on T cells (13). Therefore, it was assumed that when both increased sPD-L1 and decreased sPD-1 are present in the serum of PW, the immune reaction may be more suppressed than in offspring with decreased sPD-L1 and decreased sPD-1 in the serum. Thus, in serum from PW, the sPD-1/sPD-L1 ratio was very low and could inhibit lymphocyte proliferation in response to both autoantigen and alloantigen. In serum from offspring's CB, the sPD-1/sPD-L1 ratio was moderately low and could inhibit lymphocyte proliferation in response to autoantigen, but not significantly to alloantigen. To clarify whether the elevated sPD-L1 protein in PW's serum contributes to immune suppression, *in vitro* experiments of cytokine production assays with PHA stimulation, where an attempt was made to block specific functions of sPD-L1 in PW's serum with anti-PD-L1 antibody, were added. These experiments showed that various cytokines were increased in the presence of anti-sPD-L1 blocking antibody, suggesting that the elevated sPD-L1 protein in PW's serum may be functional, able to suppress broad immune reactions, and may thus be considered to protect the placenta and fetus from maternal immunosurveillance, at least in part.

With respect to clinical data, levels of sPD-L1 in PW showed a negative association with smoking. It has been well reported that maternal smoking impairs placental structure and function (20, 21). In addition, expression of PD-L1 mRNA in trophoblasts was reported to be increased with rising oxygen concentrations and decreased rapidly by a low oxygen concentration (5). On the other hand, administration of PD-L1 protein was demonstrated to protect against pre-eclampsia in the rat (22). Judging from these lines of evidence, it was then assumed that smoking may decrease oxygen supply to the placenta and secretion of sPD-L1 to the maternal blood stream, and the decreased sPD-L1 may further impair placental function and reduce sPD-L1 secretion to form a vicious circle. Of interest, even among non-PW, serum levels of sPD-L1 were suppressed in current smokers. PD-L1 is usually expressed on the macrophage lineage (23). Smoking impairs alveolar macrophage activation (24), which can be normalized by smoking cessation (25). In this case, smoking may be assumed to suppress sPD-L1 levels through impaired macrophage function.

There are several limitations of the present study. First, PW with major complications, such as gestational diabetes mellitus, pregnancy-induced hypertension, pre-eclampsia, preterm labor, or the need for emergent cesarean section, and who lacked high-risk fetal conditions, such as twins, intrauterine growth retardation, and congenital malformation, were excluded. Thus, associations between sPD-L1 levels of PW and these complications could not be examined. Instead, associations with smoking status and with fetal growth were examined to show that serum sPD-L1 levels can be a biomarker of placental function. Second, functional assays were added to support the hypothesis that sPD-L1 in addition to PD-L1 expressed on the surface of the placenta suppresses the maternal immune reaction to reject the placenta and fetus. It was demonstrated that the serum of PW showed stronger suppressive effects on autogenic and allogeneic immune reactions than serum of non-PW. Moreover, this suppressive effect of PW's sera was blocked using anti-PD-L1 antibody, as demonstrated by cytokine production assays. Third, MLC, which is used to evaluate the possibility of graft vs. host or host vs. graft disease in the field of bone marrow transplantation or organ transplantation, was used to examine immune reactions in the presence of PW's serum, considering the immunological paradox of pregnancy. However, there are many other types of functional assays. Fourth, since placental tissue was not collected in this cohort, for example, direct interactions between syncytiotrophoblasts and T cells could not be examined. Instead, the focus was not on membrane-bound PD-L1 molecules expressed on syncytiotrophoblasts, but on the soluble form of PD-L1 in the serum of PW. Fifth, sPD-L1 was measured at only one point in the third trimester during pregnancy. Sixth, placental PD-L1 protein expression was not measured by immunohistochemistry.

In conclusion, this is the first study to measure both sPD-L1 and sPD-1 levels in the serum of PW, as well as paired offspring, and it showed that: (1) sPD-L1 levels were very high in the serum of PW in the third trimester, but low in paired offspring; (2) sPD-1 levels were lower in both PW and offspring than in non-PW; (3) there was a strong correlation of sPD-1 levels, as well as sPD-L1 levels, between PW and offspring; (4) on MLC, ^3^H-thymidine uptake in the presence of autoantigen was strongly reduced by co-culture with sera from both PW and offspring, compared with non-PW's serum, while uptake in the presence of alloantigen was moderately inhibited by sera from PW, but it was not significantly suppressed by offspring's serum, compared with non-PW's serum; (5) adding the anti-PD-L1 blocking antibody to sera from PW raised cytokine secretion (6); and, finally, sPD-L1 levels in PW and in non-PW were suppressed by smoking.

The novel finding of this study was that PW's serum may suppress both autogenic and allogeneic immune reactions, whereas offspring's serum may suppress mainly the autogenic immune reaction and only partly allogeneic immune reactions. It was further shown that anti-PD-L1 antibody impairs the immunosuppressive effects of PW's serum, and it was suggested that elevated sPD-L1 levels in PW's sera may be functional and play, at least in part, a role in suppressing the maternal immune reaction to alloantigen, i.e., placenta and fetus. Further research is needed to confirm this.

## Author Contributions

MO and MU conceptualized and designed the study, drafted the initial manuscript, and reviewed and revised the manuscript. TK and HM collected and fixed clinical data and reviewed and revised the manuscript. MU performed experiments and analyzed data statistically. All authors approved the final manuscript.

### Conflict of Interest Statement

The authors declare that the research was conducted in the absence of any commercial or financial relationships that could be construed as a potential conflict of interest.

## References

[B1] Boussiotis VA. Molecular and biochemical aspects of the PD-1 checkpoint pathway. N Engl J Med. (2016) 375:767–78. 10.1056/NEJMra1514296 27806234 PMC5575761

[B2] Topalian SL Hodi FS Brahmer JR Gettinger SN Smith DC McDermott DF. Safety, activity, and immune correlates of anti-PD-1 antibody in cancer. N Engl J Med. (2012) 366:2443–54. 10.1056/NEJMoa1200690 22658127 PMC3544539

[B3] Brahmer JR Tykodi SS Chow LQ Hwu WJ Topalian SL Hwu P. Safety and activity of anti-PD-L1 antibody in patients with advanced cancer. N Engl J Med. (2012) 366:2455–65. 10.1056/NEJMoa1200694 22658128 PMC3563263

[B4] Veras E Kurman RJ Wang TL Shih IM. PD-L1 expression in human placentas and gestational trophoblastic diseases. Int J Gynecol Pathol. (2017) 36:146–53. 10.1097/PGP.0000000000000305 27362903 PMC5518625

[B5] Holets LM Hunt JS Petroff MG. Trophoblast CD274 (B7-H1) is differentially expressed across gestation: influence of oxygen concentration. Biol Reprod. (2006) 74:352–8. 10.1095/biolreprod.105.046581 16251499

[B6] Petroff MG Chen L Phillips TA Azzola D Sedlmayr P Hunt JS. B7 family molecules are favorably positioned at the human maternal-fetal interface. Biol Reprod. (2003) 68:1496–504. 10.1095/biolreprod.102.010058 12606489

[B7] Guleria I Khosroshahi A Ansari MJ Habicht A Azuma M Yagita H. A critical role for the programmed death ligand 1 in fetomaternal tolerance. J Exp Med. (2005) 202:231–7. 10.1084/jem.20050019 16027236 PMC2213002

[B8] Zhu X Lang J. Soluble PD-1 and PD-L1: predictive and prognostic significance in cancer. Oncotarget (2017) 8:97671–82. 10.18632/oncotarget.18311 29228642 PMC5722594

[B9] Frigola X Inman BA Lohse CM Krco CJ Cheville JC Thompson RH. Identification of a soluble form of B7-H1 that retains immunosuppressive activity and is associated with aggressive renal cell carcinoma. Clin Cancer Res. (2011) 17:1915–23. 10.1158/1078-0432.CCR-10-0250 21355078 PMC3241002

[B10] Takeuchi M Doi T Obayashi K Hirai A Yoneda K Tanaka F. Soluble PD-L1 with PD-1-binding capacity exists in the plasma of patients with non-small cell lung cancer. Immunol Lett. (2018) 196:155–60. 10.1016/j.imlet.2018.01.007 29366663

[B11] Ding Y Sun C Li J Hu L Li M Liu J. The prognostic significance of soluble programmed death ligand 1 expression in cancers: a systematic review and meta-analysis. Scand J Immunol. (2017) 86:361–7. 10.1111/sji.12596 28930374

[B12] Enninga EAL Harrington SM Creedon DJ Ruano R Markovic SN Dong H. Immune checkpoint molecules soluble program death ligand 1 and galectin-9 are increased in pregnancy. Am J Reprod Immunol. (2018) 79(2). 10.1111/aji.12795 29205636 PMC5814874

[B13] Amancha PK Hong JJ Rogers K Ansari AA Villinger F. *In vivo* blockade of the programmed cell death-1 pathway using soluble recombinant PD-1-Fc enhances CD4+ and CD8+ T cell responses but has limited clinical benefit. J Immunol. (2013) 191:6060–70. 10.4049/jimmunol.1302044 24227774 PMC3858463

[B14] Bartee MY Dunlap KM Bartee E. Tumor-localized secretion of soluble PD1 enhances oncolytic virotherapy. Cancer Res. (2017) 77:2952–63. 10.1158/0008-5472.CAN-16-1638 28314785 PMC5457316

[B15] Sorensen SF Demuth C Weber B Sorensen BS Meldgaard P. Increase in soluble PD-1 is associated with prolonged survival in patients with advanced EGFR-mutated non-small cell lung cancer treated with erlotinib. Lung Cancer (2016) 100:77–84. 10.1016/j.lungcan.2016.08.001 27597284

[B16] Niwa S Mezawa H Kobayashi N Ida H Urashima M. Inverse association between maternal 25OHD level and cord GLP-1/GIP concentrations. Pediatr Res. (2016) 79:536–42. 10.1038/pr.2015.253 26650343

[B17] Andorsky DJ Yamada RE Said J Pinkus GS Betting DJ Timmerman JM. Programmed death ligand 1 is expressed by non-hodgkin lymphomas and inhibits the activity of tumor-associated T cells. Clin Cancer Res. (2011) 17:4232–44. 10.1158/1078-0432.CCR-10-2660 21540239

[B18] Sullivan KE Cutilli J Piliero LM Ghavimi-Alagha D Starr SE Campbell DE. Measurement of cytokine secretion, intracellular protein expression, and mRNA in resting and stimulated peripheral blood mononuclear cells. Clin Diagn Lab Immunol. (2000) 7:920–4. 11063499 10.1128/cdli.7.6.920-924.2000 PMC95986

[B19] Cuzick J. A wilcoxon-type test for trend. Stat Med. (1985) 4:87–90. 10.1002/sim.4780040112 3992076

[B20] Kharkova OA Grjibovski AM Krettek A Nieboer E Odland JO. Effect of smoking behavior before and during pregnancy on selected birth outcomes among singleton full-term pregnancy: a murmansk county birth registry study. Int J Environ Res Public Health (2017) 14:E867. 10.3390/ijerph14080867 28767086 PMC5580571

[B21] Zdravkovic T Genbacev O McMaster MT Fisher SJ. The adverse effects of maternal smoking on the human placenta: a review. Placenta (2005) 26 (Suppl. A):S81–6. 10.1016/j.placenta.2005.02.003 15837073

[B22] Tian M Zhang Y Liu Z Sun G Mor G Liao A. The PD-1/PD-L1 inhibitory pathway is altered in pre-eclampsia and regulates T cell responses in pre-eclamptic rats. Sci Rep. (2016) 6:27683. 10.1038/srep27683 27277012 PMC4899740

[B23] Dong H Strome SE Salomao DR Tamura H Hirano F Flies DB. Tumor-associated B7-H1 promotes T-cell apoptosis: a potential mechanism of immune evasion. Nat Med. (2002) 8:793–800. 10.1038/nm730 12091876

[B24] Moller W Barth W Pohlit W Rust M Siekmeier R Stahlhofen W. Smoking impairs alveolar macrophage activation after inert dust exposure. Toxicol Lett. (1996) 88:131–7. 10.1016/0378-4274(96)03728-9 8920727

[B25] Hodge S Hodge G Ahern J Jersmann H Holmes M Reynolds PN. Smoking alters alveolar macrophage recognition and phagocytic ability: implications in chronic obstructive pulmonary disease. Am J Respir Cell Mol Biol. (2007) 37:748–55. 10.1165/rcmb.2007-0025OC 17630319

