# Supplementary material for: Elevated Soluble PD-L1 in Pregnant Women's Serum Suppresses the Immune Reaction
Source: Front Immunol. 2019 Feb 18;10:86. doi: 10.3389/fimmu.2019.00086 (PMC6387906; doi:10.3389/fimmu.2019.00086)
Supplement: Supplementary file 1 [file Data_Sheet_1.docx]

***Supplementary Material***

**Elevated soluble PD-L1 in pregnant women’s serum suppresses the immune reaction.**

**Mai Okuyama^1,2^, Hidetoshi Mezawa^2^, Toshinao Kawai^3^, Mitsuyoshi Urashima^1,2*^**

**^*^ Corresupondence:** Mitsuyoshi Urashima: urashima@jikei.ac.jp


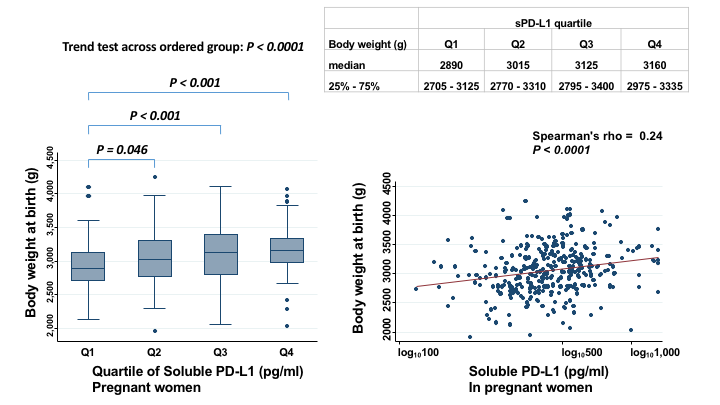


**Supplementary Figure 1. Relationship between sPD-L1 and** **offspring’s body weight at birth**

The test for trends across ordered quartiles was used to examine the relationship between offspring and sPD-L1 quartiles in PW (left panel); median and 25^th^ - 75^th^ for each quartile is shown in the upper right panel; and the correlations between serum sPD-L1 levels as a continuous variable transformed by common logarithm (log_10_) in PW and their offspring’s anthropometry are shown in the right lower panel.


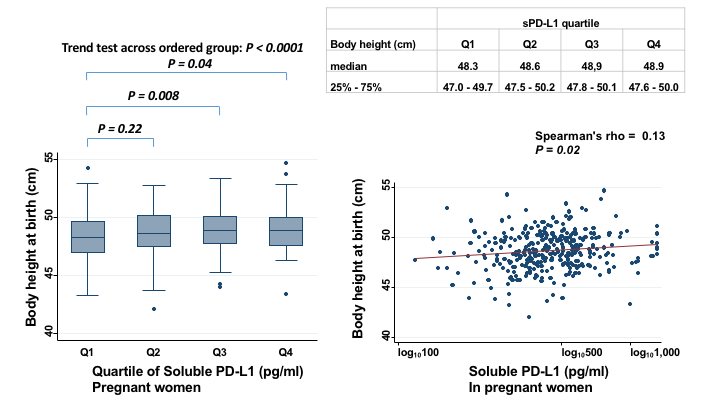


**Supplementary Figure 2. Relationship between sPD-L1 and offspring’s body height at birth**

The test for trends across ordered quartiles was used to examine the relationship between offspring and sPD-L1 quartiles in PW (left panel); median and 25th - 75th for each quartile is shown in the upper right panel; and the correlations between serum sPD-L1 levels as a continuous variable transformed by common logarithm (log_10_) in PW and their offspring’s anthropometry are shown in the right lower panel.


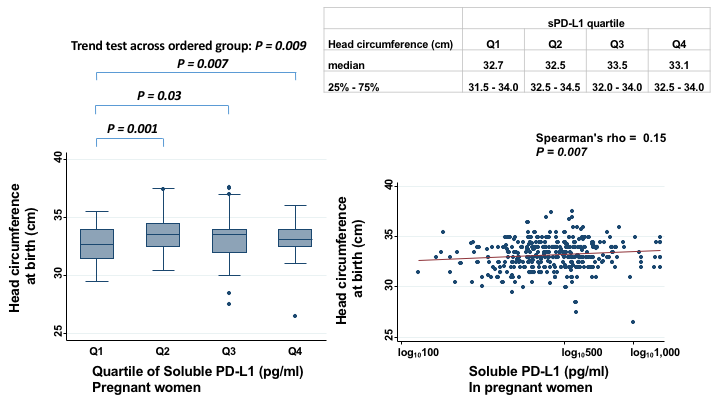


**Supplementary Figure 3.**

**Relationship between sPD-L1 and offspring’s head circumference at birth**

The test for trends across ordered quartiles was used to examine the relationship between offspring and sPD-L1 quartiles in PW (left panel); median and 25th - 75th for each quartile is shown in the upper right panel; and the correlations between serum sPD-L1 levels as a continuous variable transformed by common logarithm (log_10_) in PW and their offspring’s anthropometry are shown in the right lower panel.


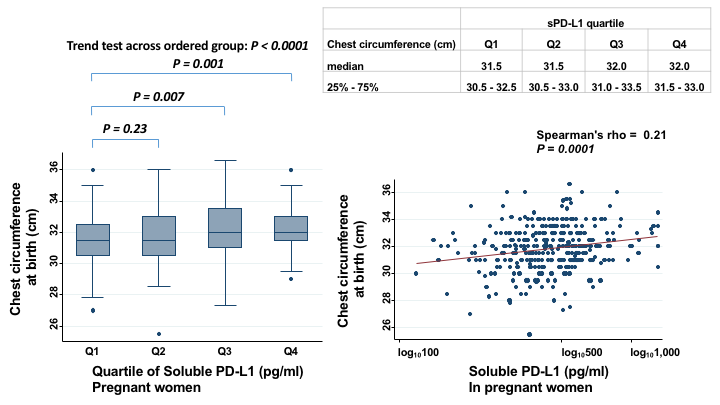


**Supplementary Figure 4.**

**Relationship between sPD-L1 and offspring’s chest circumference at birth**

The test for trends across ordered quartiles was used to examine the relationship between offspring and sPD-L1 quartiles in PW (left panel); median and 25th - 75th for each quartile is shown in the upper right panel; and the correlations between serum sPD-L1 levels as a continuous variable transformed by common logarithm (log_10_) in PW and their offspring’s anthropometry are shown in the right lower panel.
